# Coming of age: could obesity-related metabolic complications be treated by targeting senescent cells?

**DOI:** 10.3389/fcell.2025.1622107

**Published:** 2025-06-04

**Authors:** Barbora Judita Kasperova, Anna Cinkajzlova, Ludek Horvath, Petr Svoboda, Martin Haluzik, Sona Stemberkova Hubackova

**Affiliations:** ^1^ Diabetes Centre, Institute for Clinical and Experimental Medicine, Prague, Czechia; ^2^ First Faculty of Medicine, Charles University in Prague, Prague, Czechia; ^3^ Centre for Experimental Medicine, Institute for Clinical and Experimental Medicine, Prague, Czechia; ^4^ Department of Biochemistry and Microbiology, University of Chemistry and Technology, Prague, Czechia; ^5^ Institute of Medical Biochemistry and Laboratory Diagnostics, First Faculty of Medicine, Charles University and General University Hospital, Prague, Czechia; ^6^ Institute of Biotechnology, Czech Academy of Sciences, Prague, Czechia

**Keywords:** cellular senescence, senolytics, type 2 diabetes mellitus, obesity, metabolic complications

## Abstract

Aging is characterized by gradual deterioration of organ or tissue function and its ability to maintain homeostasis of the different physiological processes. This leads to the development of structural and functional alterations accompanied by an increased risk for diverse pathologies. Cellular senescence is a controlled biological process that could contribute to the development of many age-related diseases and related metabolic dysfunctions. Two major chronic diseases associated with premature accumulation of senescent cells that impose an enormous burden on global health systems are obesity and type 2 diabetes mellitus with its related complications. The purpose of this review is to highlight the links between aging, obesity, and type 2 diabetes mellitus, focusing on the role of cellular senescence in disease development and progression. Additionally, this review will discuss the potential of targeting cellular senescence as a promising therapeutic strategy for managing these interrelated diseases, therefore offering a novel approach to prevention and treatment.

## 1 Introduction

Obesity and type 2 diabetes mellitus (T2DM) represent currently major health threats worldwide owing to their rapidly increasing prevalence and debilitating long-term chronic complications such as cardiovascular disease (CVD), diabetic kidney disease (DKD), metabolic dysfunction-associated steatotic liver disease (MASLD), or diabetic neuropathy (Ogurtsova et al., 2017). Increased prevalence of T2DM is particularly evident in elderly patients, where it can affect as many as 30%–40% of the population compared with about 6%–25% of patients under 65 years of age (Wilkinson et al., 2016). Recent experimental evidence suggests cellular senescence as a potential mechanism involved in the development of insulin resistance (IR) and progression from simple obesity to T2DM.

Cellular senescence is a stress response program leading to a different cell fate defined as a stable cell cycle arrest limiting the uncontrolled proliferation of damaged cells. However, their high metabolic activity and increased production of pro-inflammatory cytokines, chemokines, growth factors, and extracellular matrix-degrading proteases, collectively called senescence-associated secretory phenotype (SASP), contribute to tissue dysfunction and activation of the immune system, which is implicated in the development of chronic inflammation and gradual tissue damage (Di Micco et al., 2021).

Senescent cells play an important role in T2DM pathogenesis via direct impact on pancreatic β-cell function, since reduced pancreatic β-cell mass and subsequent defects in insulin secretion are major factors in the pathogenesis and progression of T2DM (Sone and Kagawa, 2005). Preferential accumulation of senescent cells in visceral adipose tissue (VAT) is then associated with an inappropriate expansion of adipocytes (hypertrophy), IR, and dyslipidemia and represents the nexus of mechanisms involved in aging and age-related metabolic dysfunctions (Palmer et al., 2019). On the other hand, changes induced by long-standing, poorly controlled T2DM are linked to the accumulation of premature senescent cells in various tissues, contributing to the development of chronic irreversible complications (Palmer et al., 2015). Thus, senescence is both a cause and a consequence of obesity and T2DM.

The presence of T2DM and its complications is the major reason for the massive financial burden of the treatment of T2DM. It is estimated that therapy of diabetic complications consumes up to two-thirds of the overall T2DM treatment costs. Despite the availability of novel glucose-lowering drugs, the number of patients with T2DM and related chronic complications keeps increasing at a high rate. Current pharmacological approaches address the pathophysiological defects present in T2DM rather than preventing the processes contributing to its development (Foretz et al., 2014; Foretz and Viollet, 2015). Therapeutic targeting and elimination of senescent cells with suppression of the SASP production by senolytics may therefore be an effective strategy for a novel approach in the treatment of metabolic diseases.

## 2 Cellular senescence

Aging is characterized by a gradual deterioration of tissue function, which eventually results in organ dysfunction. In 1964, Leonard Hayflick postulated a theory that cells lose their ability to divide, but remain metabolically very active and begin processes leading to the degeneration of the cell culture (Hayflick, 1965). In contrast to the latent proliferation arrest of quiescent or terminally differentiated cells, which play a key role in maintaining homeostasis in tissues, cellular senescence represents a defence mechanism. It forces damaged and potentially cancerous cells to arrest the cell cycle and thus prevents potential transmission of damage into the next cell cycle. Although the short-term presence of senescent cells in the body may be beneficial in various settings, their long-term accumulation appears to have numerous negative effects (Hemann et al., 2001; Cristofalo et al., 2004).

**FIGURE 1:**
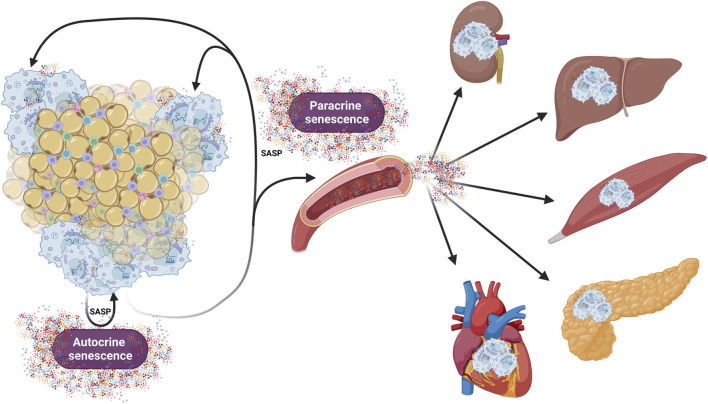
Role of autocrine, paracrine and endocrine signalling in development and maintenance of senescence in the organism. This figure illustrates how senescent cells (represented here by adipose tissue) contribute to both local and systemic senescence. Throughout the autocrine/paracrine singaling, senescent cells maintain the senescent phenotype and promote senescence in neighboring cells. Additionally, release of SASP factors into the circulation promotes senescence in distant organs, thereby contributing systemic inflammation and tissue dysfunctions. Created in https://BioRender.com.

In addition to cell cycle arrest and activation of related pathways, senescent cells undergo many other changes, such as an increase in activity of the β-galactosidase enzyme (senescence-associated β-galactosidase; SA-β-gal) due to the accumulation of lysosomes, which are involved in the proteolytic processing of damaged organelles, misfolded proteins or chromatin fragments that arise from nuclear cleavage during senescence development (Kurz et al., 2000). Development of senescence is also accompanied by significant increases in the production of numerous proinflammatory cytokines, chemokines, growth factors, and proteases, collectively termed as SASP. SASP plays an important role in autocrine/paracrine signalling and maintenance of the senescent phenotype. Because SASP results from a response to damage within a cell, one of its functions is to communicate with the immune system to remove impaired cells by mediating the activation and recruitment of both innate and adaptive immune cells (Kang et al., 2011; Soriani et al., 2009). However, long term SASP production profoundly affects the neighboring cells and contributes to the development of systemic inflammation and age-related diseases (Birch and Gil, 2020; Mosteiro et al., 2016). Studies have shown that not only direct damage, but also prolonged exposure to SASP activates responses that induce growth arrest and secondary senescence. Through paracrine signalling, molecules produced by senescent cells influence cells in close proximity. However, SASP molecules can also reach distant cells via the bloodstream as a part of endocrine signalling (Xu et al., 2018; Tsuji et al., 2022). This mechanism explains how senescent cells increase their numbers and accumulate even in tissues not affected by primary stress (Figure 1). Despite the well-known changes associated with senescence, there is no universal feature that uniquely characterizes senescent cells. This fact complicates the detection of senescent cells in the organism, which often relies on a combination of multiple markers.

**FIGURE 2:**
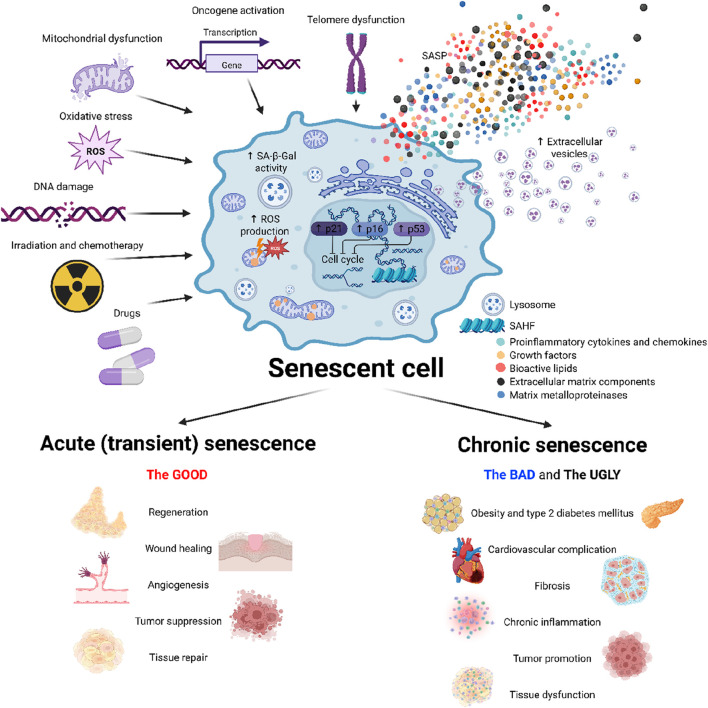
Mechanisms and impacts of cellular senescence. This figure illustrates the induction, characteristics and role of cellular senescence. Various stress factors–mitochondrial and telomere dysfunction, reactive oxygen species (ROS), DNA damage, physical stress (e.g., radiation) and chemotherapeutic agents–trigger a senescent phenotype characterized by cell cycle arrest, increased senescence-associated β-galactosidase activity (SA-β-gal), elevated reactive oxygen species (ROS) production, and transcriptional changes. Senescent cells secrete a complex combination of bioactive molecules, collectively known as a senescence-associated secretory phenotype (SASP), which influences the tissue microenvironment. Cellular senescence plays beneficial roles in physiological processes such as tissue repair, regeneration or tumor suppression. The persistent accumulation of senescent cells however promotes deleterious effects, contributing to the development of metabolic diseases, cardiovascular complication, tissue dysfunction and tumor promotion. Created in https://BioRender.com.

Cellular senescence can be triggered by a variety of internal and external factors that lead to extensive genetic and metabolic changes in the cell. As senescence can occur both as a physiological defence mechanism and as a pathological process, it is important to differentiate naturally occurring senescence and prematurely induced senescence. Natural or replicative senescence is primarily caused by progressive shortening of telomeres at each cell division during aging. When telomeres reach a critical length showing signs of DNA damage, cells activate the appropriate signalling pathways in response leading to cell cycle arrest (Bodnar et al., 1998). In contrast, premature senescence occurs independently of telomere shortening. It can be induced by physical mutagens, including ionizing and non-ionizing radiation, changes in tissue pH or temperature, nutritional changes, or hypoxia (Polonis et al., 2020; Meng et al., 2003; Patro et al., 2011; Serrano et al., 1997). However, it can also arise as a result of exposure of cells to chemicals, including therapies used in clinical practice, especially in oncology. In particular, many drugs used in oncology involve substances that damage the DNA of more than just cancer cells, leading to proliferation arrest and senescence. Other contributing factors include the accumulation of DNA damage due to increased oxidative stress from dysfunctional mitochondria, and a decline in DNA repair capacity, which may lead to genomic instability, a hallmark of both replicative and stress-induced senescence (Alsalem et al., 2024; Armstrong et al., 2014) (Figure 2).

### 2.1 Role of cellular senescence in the organism

Cellular senescence can be divided into two main forms–acute (physiological) and chronic (pathological). Acute senescence is a well-timed and controlled response of the organism to specific stimuli, including the subsequent early-onset elimination of senescent cells by the immune system. On the other hand, chronic senescence is associated particularly with the inability of the immune system to eliminate senescent cells, which leads to their accumulation in tissues and pathological changes in the organism. Acute and chronic senescence thus differ in time they remain in the body rather than in their origin (van Deursen, 2014).

In addition to preventing cancer, short-term presence of senescent cells plays an important role in reducing fibrotic tissue that accumulates during wound healing, in which connective tissue replaces normal parenchymal tissue, leading to tissue remodelling and the formation of permanent scars. Particularly, increased production of metalloproteinases by senescent cells contributes to the degradation of the extracellular matrix and reduced accumulation of connective tissue at the wound (Demaria et al., 2014; Shivshankar et al., 2012). Activation of cellular senescence also plays an important role in megakaryocyte maturation and platelet formation in bone marrow (Besancenot et al., 2010). Furthermore, the presence of senescent cells promotes remodelling and vascularization of the endometrium, which is essential for the successful implantation of the embryo and is also an integral part of the prenatal development of the kidneys and the inner ear (Munoz-Espin et al., 2013) (Figure 2).

However, the prevalence of pathological (chronic) senescence increases with aging and in subjects with weakened or suppressed immune system. During pathological senescence, tissue function is disturbed due to the prolonged presence of senescent cells manifested by reduced tissue regeneration, accumulation of fibrotic cells and loss of tissue elasticity or increased inflammation. The accumulation of senescent cells can contribute to pathologies including chronic respiratory diseases, T2DM, CVD, neurodegenerative diseases and cancer. Although senescence was originally described as an anti-cancer barrier, the production of inflammatory cytokines and chemokines may in turn contribute to the development of cancer. Increased production of SASP not only protects tumors from the immune system but also stimulates the growth and invasiveness of tumor cells and increases angiogenesis in the tumor (Salam et al., 2023). In contrast to its beneficial function in reducing fibrotic tissue during wound healing, prolonged exposure of senescent cells in the tissue may instead promote the development of fibrosis. In respiratory diseases such as idiopathic pulmonary fibrosis or chronic obstructive pulmonary disease, the presence of senescent cells has been associated with increased DNA damage, telomere shortening, inflammation, and oxidative stress, which promote fibrotic tissue in the lung and progressively decrease lung function (Schafer et al., 2017). Similarly, neurons and glial cells may be exposed to increased inflammation, DNA damage and oxidative stress as senescent cells accumulate, contributing to the development of neurodegenerative diseases such as Alzheimer’s or Parkinson’s disease (Figure 2) (Baker and Petersen, 2018).

## 3 Role of cellular senescence in obesity, T2DM and its complications

Overweight and obesity are among the most prominent health problems of the human population. In 2021 the World Health Organization (WHO) estimated that almost two billion individuals over the age of 18 were overweight, and more than 650 million were obese. The number is increasing every year also due to the rising incidence of overweight and obesity among the children population and adolescents. The reason for such a massive spread is mainly the lifestyle which combines increased intake of energy-dense food high in sugar and fat, and reduced physical activity (Obesity, 2000).

**FIGURE 3:**
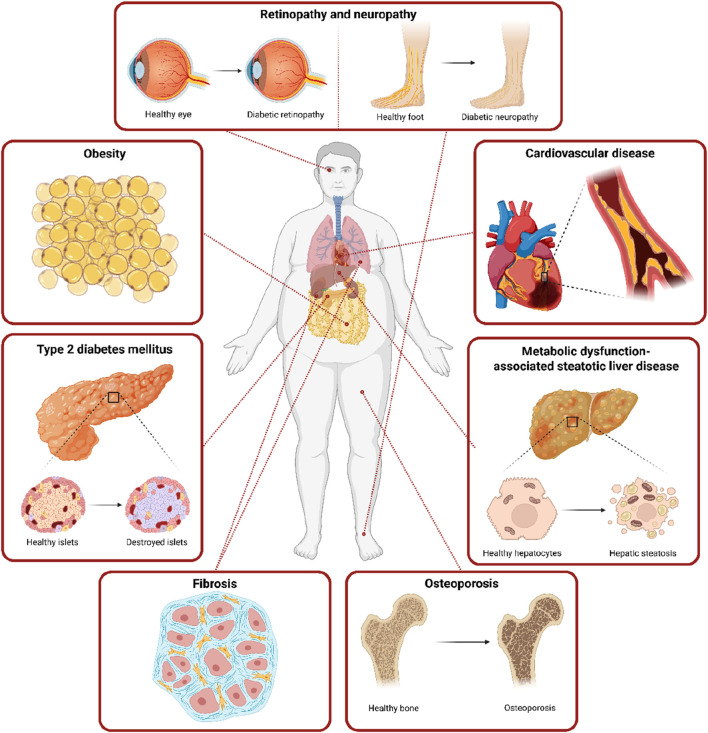
Systemic obesity- and T2DM-related complications connected with the accumulation of senescent cells. This figure highlights the systemic consequences of obesity and T2DM, with a focus on their link to the accumulation of senescent cells across multiple organs. Senescent cells through their SASP contribute to β-cell dysfunction in the pancreas, microvascular complications, hepatic steatosis and fibrosis in the liver, kidney, or lungs, impaired tissue regeneration, and complications including retinopathy and peripheral neuropathy. These pathologies underscore the systemic nature of senescence-driven dysfunction in obesity and T2DM. Created in https://BioRender.com.

Senescent cells may be involved in the pathogenesis of obesity and T2DM as a cause and consequence of metabolic changes. Chronic exposure to elevated glucose levels in patients with T2DM may contribute to the development of cellular senescence in various tissues, which ultimately contributes not only to the progression of the disease itself, but especially to the development of numerous complications including cardiorenal disease, respiratory disease, or diabetic neuropathy and retinopathy (Figure 3).

### 3.1 Obesity, T2DM and senescence

Obesity is defined by the excessive accumulation of adipose tissue in the body. The main contributors to obesity development are high caloric intake, inadequate energy expenditure, and a sedentary lifestyle, which in combination lead to the development of health complications and premature mortality. Obesity can, together with arterial hypertension, dyslipidemia, and IR, enhance the progression of metabolic syndrome and development of T2DM (Sookoian and Pirola, 2011; Zizka et al., 2024).

Adipose tissue plays a critical role in energy metabolism, thermoregulation, hormonal regulation, and metabolism of lipids and carbohydrates. Adipose tissue is also one of the largest endocrine and immune organ, as it is involved in the production and secretion of numerous hormones and cytokines, making it essential to a variety of physiological and pathophysiological processes (Coelho et al., 2013). For this reason, adipose tissue dysfunction significantly contributes to the development of metabolic diseases like obesity and T2DM by creating an inflammatory microenvironment that further accelerates cellular senescence in the body (Tchkonia et al., 2010; Kirkland and Dobson, 1997). In individuals with obesity, increased lipid deposition leads to IR, altered adipokine secretion, increased production of inflammatory molecules, and the development of cellular senescence in adipose tissue (Xu et al., 2015). Preadipocytes, the precursors of mature adipocytes (essential for fat cell turnover), tend to be the most susceptible to the development of cellular senescence. An increased number of senescent preadipocytes may contribute to a decrease in the adipogenic and lipogenic potential of adipose tissue, the development of adipose tissue dystrophy, peripheral IR, lipotoxicity, and as a consequence, the development of T2DM (Guo et al., 2007).

As pro-inflammatory SASP production increases the infiltration of immune cells into adipose tissue, senescence also contributes to both local and systemic inflammation, thus increasing the risk of metabolic complications. Monocyte Chemoattractant Protein-1 (MCP-1), one of the most secreted SASP molecules, plays an important role in the abnormal infiltration of macrophages into adipose tissue and their disproportionate differentiation into pro-inflammatory M1 macrophages (Covarrubias et al., 2020). The accumulation of senescent cells and SASP in adipose tissue also ultimately leads to the accumulation of free fatty acids, which, in turn, exacerbates cellular dysfunction and worsens the progression of T2DM and its complications (Wiley and Campisi, 2021).

The contribution of senescent cells to the development and progression of metabolic diseases differs significantly between adipose depots. VAT exhibits a higher burden of senescent cells compared to subcutaneous adipose tissue (SAT). SASP produced by senescent cells in VAT disrupts local insulin signaling, promotes immune cell infiltration, and induces systemic inflammation, exacerbating metabolic dysfunction. Proximity of VAT to the liver via the portal circulation allows these inflammatory mediators to increase hepatic insulin resistance and impair glucose metabolism (Villaret et al., 2010; Fox et al., 2007). In contrast, SAT tends to accumulate fewer senescent cells and exhibits a milder SASP profile. It is also better vascularized and less inflamed adipose depot. Senescent cell burden has, therefore, a less direct impact on metabolic health (Ibrahim, 2010). Several studies have shown that higher senescent markers in VAT correlate more strongly with poor glycemic control and T2DM risk than those in SAT (Alessio et al., 2020; Lefevre et al., 2021). These findings highlight the importance of adipose tissue depot-specific senescence in obesity-related metabolic diseases.

While VAT and SAT are the most metabolically harmful depots when senescent, other white adipose depots - especially perivascular adipose tissue and bone marrow adipose tissue - also contribute to systemic disease through local inflammation, paracrine signaling, and endocrine disruption. Both depots are emerging targets in age- and obesity-related conditions, including cardiovascular disease, bone loss, and immune dysfunction (Tencerova et al., 2019; Kim et al., 2020).

Unlike white adipose tissue, brown adipose tissue (BAT) plays a protective role in energy balance through thermogenesis, primarily mediated by uncoupling protein 1 (UCP1). However, the buildup of senescent cells impairs this function and contributes to metabolic dysfunction. Senescent cells in BAT exhibit mitochondrial dysfunction and reduced UCP1 expression, which leads to impaired thermogenic capacity and reduced energy expenditure. This energy imbalance can promote weight gain and exacerbate insulin resistance (Feng et al., 2023). Furthermore, senescent brown adipocytes and their precursors develop a hostile local environment that impairs the differentiation and function of new brown adipocytes and drives inflammation-induced “whitening” of BAT, where BAT takes on white fat-like properties and loses its thermogenic function (Pan et al., 2021). Moreover, impaired BAT thermogenesis and inflammation contribute to broader metabolic disturbances, including glucose intolerance, hyperlipidemia, and hepatic insulin resistance. Experimental studies in mice have shown that the elimination of senescent cells in BAT using senolytic therapies can restore thermogenic gene expression, reduce inflammation, and improve insulin sensitivity (de Oliveira Silva et al., 2025).

The metabolic changes and systemic inflammation in individuals with obesity play a critical role in promoting the formation and subsequent accumulation of senescent cells throughout the body. These senescent cells contribute not only to the progressive deterioration of tissue function but also to the onset and exacerbation of various chronic complications of obesity, including T2DM, diabetic neuropathy, DKD or cardiovascular complications. Senescent cells are therefore considered part of the ‘pathogenic loop’ in subjects with obesity and T2DM (Palmer et al., 2015).

### 3.2 Senescence-associated diabetic kidney disease

Of the long-term complications of obesity and T2DM, DKD imposes the highest-burden both in terms of financial cost and the effects on daily life. DKD is a common and potentially devastating disorder characterized by progressive loss of kidney function and persistent renal fibrosis, leading to the destruction of kidney parenchyma and renal failure. Excessive renal fibrosis progressing to DKD affects approximately 40% of patients with obesity or T2DM, and this percentage increases with the age of the patient (Nordheim and Geir Jenssen, 2021). The pathology of DKD is significantly affected by oxidative stress, which is more prevalent in aging kidneys (Zhou et al., 2019; Gomes et al., 2009). Advanced glycation endproducts (AGEs) and advanced oxidative protein products are formed during senescence and contribute to the faster development of DKD under hyperglycemic conditions. AGEs and advanced oxidative protein products then interact with AGEs receptors, followed by activation of the transforming growth factor β (TGF-β), nuclear factor kappa-light-chain-enhancer of activated B cells (NK-κB), mitogen-activated protein kinase (MAPK), or NADPH oxidase pathways (Gomes et al., 2009; Chen YY. et al., 2019; Liu et al., 2015). This results in the secretion of many cytokines and growth factors, which eventually cause damage to renal tissue and enhance premature kidney aging.

Senescent cells can be detected in various renal tissue compartments with the highest prevalence in the proximal tubular epithelium, glomeruli, or endothelium (Sis et al., 2007; Verzola et al., 2008). Source, intensity, and duration of the stress stimuli determine the location and type of senescent cells. In general, elevated level of senescent cells in renal tissue correlates with the severity of DKD. Similarly, in kidney transplantation, most frequently occurring in patients with obesity and T2DM, the prognosis of graft survival depends on the age of the transplanted organ. It was described that the level of the cellular senescence marker p16^INK4a^ observed in the preimplantation biopsy predicts the functionality of the implanted graft (Sofue et al., 2018).

T2DM additionally contributes to pathological activation of the renin-angiotensin-aldosterone system (RAAS) and subsequently leads to the progression of fibrosis and hypertension, which is further exacerbated by the accumulation of senescent cells. Dysregulation of RAAS and the impairment in renal function may also adversely impact CVD development due to hemodynamic changes and induction of cardiac fibrosis (AlQudah et al., 2020; Gabbin et al., 2022; Rex et al., 2023).

### 3.3 The role of senescence in the development of cardiovascular diseases

A combination of obesity and T2DM is a major risk factor for the development of CVD. Overexpression of pro-inflammatory cytokines (tumor necrosis factor α (TNF-α), interleukin 1 (IL-1), interleukin 6 (IL-6), leptin, MCP-1, plasminogen activator inhibitor 1(PAI-1), fibrinogen, angiotensin) contributes to the development of IR and T2DM. Subsequent increase in systemic inflammation and lipid accumulation have damaging effects on blood vessels and leads to endothelial dysfunction and accelerated atherosclerosis with subsequent increase in the risk of cardiovascular complications and heart failure, cardiomyopathy, and ischemia (Pradhan et al., 2001). Numerous studies have shown that high amounts of circulating non-esterified fatty acids and pro-inflammatory cytokines contribute to IR by phosphorylation of glucose transporter and subsequently impaired glucose transport in patients with obesity contributing to development/progression of T2DM (Kahn et al., 2006). High levels of blood glucose together with endothelial cells over-stimulated by insulin result in activation of pro-atherogenic pathway and the development of CVD (Chen PY. et al., 2019).

High glucose levels promote a senescent state in endothelial progenitor cells and vascular endothelial cells, both of which play an important role in the protection against endothelial damage and prevention of atherosclerosis (Kuki et al., 2006; Zhong et al., 2010). Similarly, epicardial adipose tissue-derived mesenchymal stem cells were driven towards accelerated senescence based on increased circulating glucose levels (Zhang et al., 2017). This is especially of interest in the light of the data indicating epicardial adipose tissue plays a direct role in the development of coronary atherosclerosis and myocardial dysfunction (Matloch et al., 2018). Moreover, experimental data show that elimination of senescent cardiac progenitor cells abrogated the SASP and increased the number of proliferating cardiomyocytes (Lewis-McDougall et al., 2019), while also being associated with improved cardiac diastolic function in experimental settings (Palmer et al., 2019).

In addition to CVD development, accumulation of fibrotic tissue in the heart represents another cardiac pathology in patients with obesity and T2DM (Wang et al., 2019; Cao et al., 2018). Transient fibrosis in myocardium appears as a repair mechanism that maintains the integrity of myocardial wall. Long-term presence of fibrotic cells however leads to impaired function of cardiomyocytes and overall dysfunction of myocardium. Accumulation of senescent cells in the heart due to the high level of glucose and insulin in blood together with low-grade systemic inflammation and a compromised immune system in patients with obesity and T2DM support cardiac fibrosis and hypertrophy through numerous pathophysiological pathways. This includes an excessive production of extracellular matrix in the heart, where senescent myofibroblasts are the main source of collagen secretion. Especially collagen types IV, VI, and VII were found to be involved in the development of cardiac fibrosis. Similarly, production of cytokines such as insulin growth factor 1 (IGF1), epidermal growth factor (EGF), colony-stimulating factor 2 (CSF2), and TGF-β by cardiac senescent cells strongly enhance fibroblast proliferation and transformation crucial in fibrotic processes. Furthermore, obesity and T2DM are also associated with elevated oxidative stress, which contributes to the myofibrosis development by disrupting balance between ROS and antioxidants in the body. Additionally, the formation and accumulation of AGEs due to obesity and IR may promote collagen deposition and increase myocardial stiffness, ultimately leading to fibrosis development (Gevaert et al., 2017; Abdellatif et al., 2022; Zhu et al., 2013).

### 3.4 The role of cellular senescence in the development of MASH, MASLD, and liver steatosis

T2DM is a major metabolic disorder that along with obesity significantly contributes to the development and progression of liver steatosis, MASLD, and its inflammatory and fibrotic form, metabolic dysfunction-associated steatohepatitis (MASH). While MASLD affects approximately 25%–30% of the general population, its prevalence rises to 55%–80% in those with T2DM. Among MASLD patients, progression to MASH is present in 37%–45% of subject with T2DM, compared to 10%–20% of subjects without T2DM (Le et al., 2025; Mittal et al., 2024; Younossi et al., 2024). IR promotes hepatic lipid accumulation by enhancing *de novo* lipogenesis, impairing fatty acid oxidation, and reducing lipid export from the liver. Chronic hyperglycemia and associated metabolic stress also contribute to hepatocellular injury, oxidative stress, and inflammation, creating a pro-steatotic and pro-fibrotic environment. An increasingly recognized contributor to this process is the accumulation of senescent cells within the hepatic environment (Perry et al., 2014; Kiss et al., 2020).

In hepatic steatosis, senescent hepatocytes promote lipid accumulation by disrupting metabolic homeostasis. Specifically, they exhibit increased expression of fatty acid transporters such as CD36, promoting excessive fatty acid uptake, while simultaneously downregulating pathways involved in mitochondrial beta-oxidation and lipid export (Ogrodnik et al., 2017). This metabolic shift leads to intracellular lipid accumulation and the formation of macrovesicular steatosis. Moreover, senescent hepatocytes demonstrate impaired responsiveness to insulin, further exacerbating hepatic IR and enhancing *de novo* lipogenesis through upregulation of transcription factors such as SREBP-1c. These alterations create a lipid-rich, pro-inflammatory microenvironment that fosters disease progression. As the disease advances from steatosis to steatohepatitis (MASH), the paracrine effects of senescent cells become more apparent. Senescent hepatocytes secrete a pro-inflammatory secretome that includes cytokines such as IL-6, IL-1, TNF-α, and chemokines like MCP-1, which recruit immune cells, particularly Kupffer cells and monocyte-derived macrophages, into the liver parenchyma. The immune cell activation and cytokine release contribute to hepatocellular injury, ballooning, and lobular inflammation hallmark features of MASH (Bonnet et al., 2022; Du et al., 2025).

In addition to hepatocytes, other liver cell populations, including hepatic stellate cells (HSCs) and endothelial cells, may also undergo senescence in MASLD. Senescent HSCs, although initially less fibrogenic due to cell cycle arrest, secrete SASP factors that include pro-fibrotic mediators such as TGF-β, connective tissue growth factor, and matrix metalloproteinases (MMPs). These factors promote extracellular matrix remodeling and stimulate neighboring non-senescent HSCs to adopt a fibrogenic phenotype. Over time, this contributes to progressive deposition of collagen and hepatic fibrosis, a defining feature of advanced MASH. Experimental models provide strong support for the role of cellular senescence in liver disease progression. Mouse models of diet-induced steatosis and fibrosis have shown increased hepatic expression of senescence markers such as p16^INK4a^ and p21^CIP1^, particularly in hepatocytes and HSCs. Notably, pharmacological or genetical ablation of senescent cells or caloric restriction in these models reduces hepatic lipid accumulation, inflammation, and fibrosis, demonstrating a causal role for senescence in disease pathogenesis (Ogrodnik et al., 2017; Du et al., 2025; Nehme et al., 2023). Given the central role of senescence in amplifying metabolic dysfunction, inflammation, and fibrosis, it represents a novel and attractive target for therapeutic intervention in MASLD and MASH.

### 3.5 Cellular senescence in pancreatic β-cells

As mentioned above, excessive adipose tissue accumulation in the body accelerates cellular senescence and leads to impairment in insulin secretion, chronic inflammation, and immune system dysfunction. Healthy pancreatic β-cells and sufficient insulin secretion are the key factors in maintaining euglycemia, preventing the development of T2DM. Senescent β-cells are therefore considered to be contributors to the onset of T2DM. The accumulation of senescent pancreatic β-cells may explain functional changes in pancreas during aging including mitochondrial dysfunction of cells, accumulation of AGEs, lower levels of glucose transporter 2 (GLUT2), reduced telomere length, or downregulation of β-cell markers [pancreatic and duodenal homeobox 1 (PDX1), Insulin 1 (Ins1), v-maf musculoaponeurotic fibrosarcoma oncogene family, protein A (MafA) (Aguayo-Mazzucato et al., 2019).

As mitochondria play an important role in maintenance of cellular homeostasis, mitochondrial dysfunction may have a major impact on β-cells and their stimulus-secretion coupling (Cree et al., 2008; Supale et al., 2013). Mitochondrial metabolism of β-cells is closely associated with insulin exocytosis. ATP generated as a product of pyruvate metabolism in mitochondria plays important role in insulin exocytosis via cellular depolarization (ATP-dependent K^+^ channel, voltage-gated Ca^2+^ channels, sodium, and chloride channels) (Maechler et al., 2006). At the same time, mitochondria represent the primary source of ROS. Atypical production of ROS in mitochondria may contribute to their dysfunction due to aging (Dai et al., 2014).

In recent studies it has been reported that a specific subpopulation of β-cells expresses p16^INK4a^, p53BP1, IGF1 receptor, and SA-β-gal known to be increased during T2DM. Senescent β-cells attenuate forkhead box M1 (FOXM1) regulator of cell cycle and proliferation and expression of PDX1, which is responsible for β-cell division and maturation (Krupczak-Hollis et al., 2003; Reers et al., 2009; Ihm et al., 2007). Except cell cycle regulation it has been shown that senescent β-cells downregulate genes involved in glucose metabolism, incretin signaling pathways, and insulin synthesis (Aguayo-Mazzucato et al., 2017). However, senescent β-cells display higher basal insulin secretion similarly to cells in aged subjects pointing increased insulin accumulation in these cells. Similarly as for adipose tissue, accumulation of senescent β-cells correspond to increased production of SASP in pancreas, which contributes to overall systemic inflammation detected in patients with obesity and T2DM. Thus, transcriptional and proteomic changes caused by the senescent state in β-cells are associated with an age-dependent decline in physiological function and cell proliferation which strongly contribute to the development of metabolic disorders and increase the risk of developing pancreatic tumors (Aguayo-Mazzucato et al., 2019).

### 3.6 Senescence-associated diabetic neuropathy

Diabetic neuropathy is a prevalent and debilitating complication of T2DM, with significant consequences for the quality of life in individuals. This unique neurodegenerative disorder of the peripheral nervous system arises from a multifactorial etiology involving elevated glucose levels, metabolic disturbances, and increased levels of inflammatory factors in individuals with T2DM. These changes not only impair neurogenesis, but also contribute to the accumulation of senescent cells, particularly glial cells, Schwann cells and neurons, within the nervous system. The relationship between diabetic neuropathy and cellular senescence is complex. The accumulation of senescent cells over time can impair tissue regeneration and repairing processes, furthermore preventing the recovery from diabetic neuropathy. Moreover, presence of senescent cells may disrupt the delicacy of cellular microenvironment balance, which negatively affect neighboring cells through the oxidative stress and production of cytokines (including IL-1, IL-6, TNF-α, TGF-β). This results in axonal degeneration, slower impulse conduction, and impaired peripheral tissue repair (Ogrodnik et al., 2019).

One of the main clinical manifestations of diabetic neuropathy is in the pathogenesis of diabetic foot syndrome, a condition characterized by nerve damage and poor blood supply to the limbs, which significantly increases the risk of ulceration and amputation in individuals with T2DM. Promising therapeutic approaches, such as allogeneic transplantation of mesenchymal stem cells (MSCs), have demonstrated improvements in wound healing and reductions in amputation rates. Nonetheless, individuals with T2DM often exhibit reduced numbers of circulating MSCs, alongside impaired proliferative capacity and survival. These limitations are exacerbated by the chronic pro-inflammatory environment in T2DM, which promotes MSCs replicative senescence and impairs their ability to differentiate and expand (Fijany et al., 2019). This accumulation of senescent MSCs not only compromises tissue regeneration mechanisms but also sustains a pro-inflammatory environment that further impairs the healing process in chronic wounds.

### 3.7 Senescence-associated diabetic retinopathy

As previously mentioned, cellular senescence is involved in multiple complications arising from T2DM, including diabetic retinopathy (DR). DR is characterized by retinal vascular damage resulting in vision impairment and, if not treated, significant vision loss and blindness (Leasher et al., 2016). DR is divided into two main types–non-proliferative and proliferative DR. The pathology of DR is associated with both neurodegenerative and microvascular changes in the retina. The risk of DR development is strongly linked to T2DM, hyperglycaemia, and hypertension. These factors contribute to microvascular damage, as well as retinal neurodegeneration through elevated glucose levels, inflammation, and ROS production, and subsequent accumulation of senescent cells (Song et al., 2019).

Physiologically, endothelial cells function as a selective barrier within a vascular network regulating blood flow, supporting oxygen and nutrients exchanged with the retina. However, chronic hyperglycaemia negatively affects retinal endothelial cells, causing vascular injury and accumulation of senescent cells, which contribute to pathological angiogenesis by SASP production (Liao et al., 2024). In the retina, senescent endothelial cells secrete pro-inflammatory cytokines, growth factors, and proteases, including IL-6, IL-8, IL-1β, intercellular adhesion molecule 1 (ICAM-1), vascular endothelial growth factor (VEGF), and MMPs (Miyamoto et al., 1999; Joussen et al., 2004). These factors induce further cellular senescence and local inflammation, contributing to microvascular dysfunction, abnormal blood neovascularization, and further spread via paracrine signaling (Oubaha et al., 2016).

### 3.8 Diabetes-related pulmonary fibrosis

Senescence-associated pulmonary fibrosis, characterized by progressive scarring and stiffening of lung tissue, commonly found in patients with advanced type 1 diabetes mellitus and T2DM, represents one of the aspects contributing to the severity of diabetes (Schafer et al., 2017; Barnes, 2017). Increased presence of pulmonary lipofibroblasts located in the alveolar interstitium of patients with diabetes demonstrates the ability to transdifferentiate into myofibroblasts. These premature senescent cells play an integral part of pulmonary fibrosis development [reviewed in Kruglikov and Scherer (2020)]. Except for cytokine storm, the pulmonary lipofibroblasts, an adipocyte-like cells, promote fibrosis by production of SASP, especially collagen and fibronectin, which induce DNA damage and matrix remodeling (Sabin and Anderson, 2011). The functional and structural changes in the lungs are derived at the biochemical level. Oxidative stress could be considered as an initiation factor of various diabetic complications, like diabetes-induced pulmonary fibrosis. Products of oxidative stress (ROS and reactive forms of nitrogen) directly damage lung cells. Oxidative stress activates immune cells and stimulates secretion of numerous pro-inflammatory cytokines, such as TNF-α, IL-1, PAI-1, and pro-fibrotic cytokines, like TGF-β (Bringardner et al., 2008; Sgalla et al., 2018). TNF-α may be a crucial mediator in Western diet-induced IR. It was described that knocking out or blocking TNF-α provides protection against pulmonary fibrosis in mice induced by silica and bleomycin (Ortiz et al., 1998; Ortiz et al., 1999). Similarly to TNF-α, PAI-1 seems to play an important role in the pathological formation of connective tissue. Multiple studies have shown that a lack of PAI-1 protects the lung tissue from excessive accumulation of fibrin (Shioya et al., 2018). Levels of PAI-1 increase under inflammatory conditions, as in metabolic syndrome or obesity. This leads to the formation of fibrin deposits and the development of fibrotic tissue (Nawaz and Siddiqui, 2022). Finally, activation of TGF-β signaling pathway may be the linking mechanism between cellular senescence and diabetes-related pulmonary fibrosis. TGF-β, as a pro-fibrotic cytokine, is an important mediator in pulmonary fibrosis, which induces the overproduction of extracellular collagens. The presence of senescent cells may impair the ability of lung stem/progenitor cells to regenerate, further contributing to the progression of fibrosis (Chen et al., 2020).

## 4 Reduction of senescence in the management of obesity, T2DM, and its complications

Considering the crucial role of cellular senescence in pathological processes linked to obesity and T2DM, focusing on the underlying mechanisms of aging may provide a new therapeutic approach to the treatment of metabolic diseases and prevention of their complications. Senolytics, substances that specifically remove senescent cells from the organism, could therefore represent a transformative shift in the approach to both the prevention and the treatment of obesity, T2DM, and their complications.

### 4.1 Senolytics

The concept of senolytics was first described in 2004 when several studies demonstrated that both impaired growth hormone signalling and metabolic interventions, such as caloric restriction, reduced the accumulation of senescent cells in rodents (Shimokawa et al., 2003; Ning et al., 2013; Wang et al., 2010) and extended their life span (Aguiar-Oliveira and Bartke, 2019). This led to the hypothesis that targeted removal of senescent cells may alleviate diseases associated with aging, including metabolic diseases and their complications. Nowadays, several senolytic agents are currently being tested in preclinical studies or clinical trials. Most of them target specific proteins or pathways that protect senescent cells from cell death, such as B-cell lymphoma two family (BCL-2) proteins, phosphoinositide 3-kinase (PI3K)/AKT pathway, mammalian/mechanistic target of rapamycin (mTOR), or sirtuin 1 (SIRT1).

One of the most promising senolytic agents is the combination of dasatinib and quercetin (D + Q). Dasatinib, which has been approved for clinical use by the FDA in the United States since 2006, is a tyrosine kinase inhibitor that effectively suppresses cell proliferation and migration, activates apoptosis, and is used in the treatment of cancer such as chronic myeloid leukemia and gastrointestinal stromal tumors. Quercetin is a natural flavonoid, found in many fruits and vegetables, with diverse biological activities. These substances alone have not shown a significant senolytic effect on their own. However, their combination shows a significant effect in the elimination of senescent cells by targeting the PI3K/AKT pathway (Zhu et al., 2015). In preclinical studies, the effect of senescent cell elimination by D + Q on reducing obesity-induced metabolic dysfunction has been demonstrated (Palmer et al., 2019). In this context, the effect of these agents on the reduction of MASLD is being tested. However, the D + Q combination also effectively works on other complications associated with obesity and T2DM, including idiopathic pulmonary fibrosis. A positive effect of D + Q on the reduction of fibrotic tissue and improvement of respiratory function has been demonstrated during phase 1 clinical trial (NCT02874989) (Nambiar et al., 2023). Other studies show a beneficial effect of D + Q on the reduction of kidney damage, where the removal of senescent cells leads to improved function of this organ (Zhu et al., 2024). The effect of D + Q treatment in subjects with chronic kidney damage is currently tested in phase 2 clinical trial (NCT02848131). The effect of D + Q on reducing neurodegenerative disease is being tested in phase 1/2 clinical trial (NCT04063124).

Fisetin is another natural flavonoid compound that has shown similar antioxidant properties as quercetin. The oral or dietary administration of fisetin reduces markers of senescence and SASP in multiple tissues (Yousefzadeh et al., 2018). In preclinical research (Wissler et al., 2021), fisetin has been shown to reduce senescent cells and SASP induced chronic inflammation in aged mice, thereby extending their lifespan (Yousefzadeh et al., 2018). Further studies suggest that fisetin alleviates diabetic nephropathy (Dong et al., 2022), and augments antioxidant activity as well as prevents inflammation in cardiac tissue of animals with induced prediabetes (Althunibat et al., 2019). In this context, a phase 2 clinical trial is underway to assess the effect of senescent cell elimination on improving cardiovascular function (NCT06133634).

Metformin is an approved and widely used glucose-lowering drug for treating T2DM. It inhibits glucose absorption, increases peripheral insulin sensitivity, and reduces glucose synthesis in the liver. In addition to glucose-lowering properties, metformin positively impacts metabolic and cellular processes associated with age-related conditions such as inflammation and cellular senescence (Martin-Montalvo et al., 2013). One of the described mechanisms, how metformin reduces cellular senescence, involves the inhibition of mitochondrial complex I (CI), which is involved in the cellular energy metabolism. This inhibition activates AMP-activated protein kinase (AMPK), which subsequently activates cell death pathways (Owen et al., 2000). Inhibition of CI and subsequent activation of AMPK also increases the production of nicotinamide adenine dinucleotide (NAD+), a cofactor necessary for SIRT1 activity. SIRT1 activation has been shown to improve mitochondrial metabolism/function and to protect against age-related diseases such as CVD, neurodegenerative disorders, and cancer (Owen et al., 2000; Zheng et al., 2020). The Metformin In LongEvity Study (MILES) (Kulkarni et al., 2018) and Targeting Aging with MEtformin (TAME) (NCT02432287) (Barzilai et al., 2016) clinical trials aim to demonstrate that metformin specifically affects human aging while slowing the development of aging-related complications. Other aims of the studies include measuring physical and cognitive function and quality of life of participants. Although metformin shows effects in reducing cellular senescence, it was not originally designed as a senolytic agent and its senolytic effects may be less specific compared to agents designed to primarily target senescent cells. Also, the optimal dose and duration of treatment to achieve senolytic effect are still not established. Determining the right balance between efficacy and safety is essential, as metformin can have gastrointestinal side effects and, in rare cases can cause lactic acidosis and must be discontinued in subjects with impaired renal function (Raicevic and Jankovic, 2023).

Glucagon-like peptide-1 (GLP-1) agonists are a group of drugs commonly used in the treatment of T2DM and obesity. They mimic the action of endogenous GLP-1, a hormone that stimulates insulin secretion, suppresses glucagon secretion, slows gastric emptying, and promotes satiety. Although their primary role is to improve glucose control, emerging evidence suggests that GLP-1 agonists may also have a beneficial effect on cellular aging. Although GLP-1 agonists are not considered senolytic agents, some studies show that GLP-1 treatment helps reduce senescent cells in the organism (Kimura et al., 2009; Miao et al., 2013). The mechanism by which they reduce senescence is not fully understood, however, they role in increased susceptibility of senescent cells to cell death is discussed (Peng et al., 2022).

Dual GLP-1 and glucose-dependent insulinotropic peptide receptor agonist tirzepatide is a novel promising drug for T2DM treatment and body weight loss (Wadden et al., 2023). The beneficial effects of tirzepatide treatment are associated with a significant decrease in the infiltration of pro-inflammatory M1-type macrophages within adipose tissue, inflammation associated with obesity, and improvement in IR (Xia et al., 2024). Although the effect of tirzepatide on cellular senescence remains unconfirmed, it may positively influence the inflammatory environment in the body and help to reduce obesity- and T2DM-associated senescence burden.

Sodium-Glucose Transport Protein 2 (SGLT2) inhibitors, also called gliflozins, are another widely used treatments for T2DM. However, this class of drugs has been shown to have numerous clinical benefits beyond glucose lowering such as decreasing the risk of cardiovascular and renal complications, including reduction of cellular senescence. It was described, that dapagliflozin reduces the incidence of senescent cells in the kidneys of diabetic animals by increasing the amount of ketone bodies in plasma and subsequently reducing oxidative stress, which plays an important role in the regulation of aging (Kim et al., 2021). Empagliflozin reduces senescence of cardiac stromal cells and improves cardiac function in a mouse model of diabetes through an increase in insulin-regulated AKT kinase signalling (Madonna et al., 2020; Maejima, 2019). In addition to the heart and kidney, the positive effect of SGLT2 inhibition on reducing senescence has also been found in the liver and adipose tissue (Trnovska et al., 2021).

Rapamycin, also known as sirolimus, is an mTOR inhibitor with extensive application in human medicine, including its use as an immunosuppressive or cytostatic drug. Rapamycin has been intensively studied recently for its senolytic effects (Wang et al., 2017; Sasaki et al., 2020). Rapamycin selectively activates cell death in senescent cells by inhibiting the mTOR protein, which plays an important role in their survival (Harrison et al., 2009).

Resveratrol is a natural polyphenol compound found in red grapes, berries, peanuts, etc. Due to its anti-inflammatory and antioxidant effects, resveratrol mitigates the detrimental effects of high-calorie diet in mice modulating known longevity-associated pathways, enhancing mitochondrial function and improving metabolic health independently of weight loss (Baur et al., 2006). While the exact mechanism remains unclear, the beneficial effect of resveratrol may involve the AMPK/SIRT1 pathway, which promotes the clearance of impaired/senescent cells (Park et al., 2012). These findings suggest that resveratrol may support metabolic health and delay age-related decline.

Nicotinamide mononucleotide (NMN) is a precursor of NAD+ that plays a key role in various cellular processes, including energy metabolism, DNA repair, and regulation of gene expression. With increasing age, cells tend to decrease NAD+ levels, which is associated with the accumulation of senescent cells and deteriorating health due to tissue dysfunction. Preclinical studies in animal models have shown promising results, suggesting that NMN supplementation may improve mitochondrial function and cellular metabolism, reduce inflammation, mitigate tissue damage, and extend lifespan (Mills et al., 2016; Yoshino et al., 2011). Current clinical trials show that oral administration of NMN is generally safe, and although only a limited number of markers have been studied, the results suggest that NMN has potential as an anti-aging agent (Okabe et al., 2022). However, dosage, duration of administration, and toxicological parameters still need to be resolved before NMN can be used for clinical purposes.

Nicotinamide riboside (NR) has recently become one of the most studied NAD+ precursors due to its numerous potential health benefits. NR has confirmed its efficacy in the treatment of cardiovascular, neurodegenerative, and metabolic disorders in numerous preclinical studies. Although the exact mechanism is unknown, NR has demonstrated a positive effect on longevity by reducing the amount of circulating inflammatory cytokines, thus modulating the aging process (Mehmel et al., 2020).

MitoTam is a potential anti-cancer agent (Rohlenova et al., 2017) with a determined senolytic effect. Treatment with MitoTam effectively reduces oxidative phosphorylation via targeting CI (in nanomolar concentration compared to millimolar concentrations used for metformin) and mitochondrial polarization in senescent cells, which results in dysfunction of ATPase and collapse of mitochondrial integrity and function (Hubackova et al., 2019). Although MitoTam represents mitochondria-targeted tamoxifen, the presence of a specific triphenylphosphonium vector provides the preferential uptake into highly polarized mitochondria detected in cancer and senescent cells, which changes the mechanism of action of this compound. This results, unlike tamoxifen, in preferential regulation of mitochondrial biogenesis and integrity independently of estrogen receptor. Similarly to D + Q combination, the observed glucose-lowering effect of MitoTam is linked to an improvement of T2DM-related hormone profiles and suppressed adipogenesis in adipose tissue, which represents one of the major regulators of metabolic changes in patients with obesity and T2DM. Moreover, MitoTam treatment reduced lipid accumulation in the liver, which correlates with the elimination of senescent cells by ferroptosis (Vacurova et al., 2025). Lower senescent cell burden in various tissues also results in a lower level of circulating inflammatory mediators that enhance metabolic dysfunction. Furthermore, the prolonged effect of MitoTam on weight reduction in comparison with tamoxifen, where only a transient effect followed by over-compensation results in increased fat mass and development of T2DM was described (Vacurova et al., 2022).

### 4.2 Non-pharmacological reduction of cellular senescence

Since adipose tissue represents the biggest depository of senescent cells, especially in patients with obesity and T2DM, its reduction by physical activity has been shown to reduce the presence of senescent cells not only in this tissue but also throughout the body. In addition, physical activity stimulates the production of antioxidants and activates DNA and mitochondrial repair mechanisms leading to a reduction in oxidative stress and inflammation. Physical activity also promotes adipocyte differentiation, lipid metabolism, and the secretion of anti-inflammatory adipokines, all of which contribute to the prevention of metabolic disorders. In addition, physical activity supports the mechanisms responsible for the removal of senescent cells by immune cells (El Assar et al., 2022). Incorporating regular exercise into a lifestyle is therefore an effective strategy for maintaining adipose tissue health and attenuating the negative effects of aging on metabolic function.

Bariatric surgery (also known as metabolic surgery, reflecting its extremely beneficial metabolic effects in patients with obesity) is currently the most effective approach for obesity and T2DM treatment, leading to significant and long-term weight loss and improvement in the metabolic complications of obesity. Improvements in parameters such as insulin sensitivity, glucose tolerance, lipid profile, and inflammation, contribute to a reduction of cellular stress and mitigate senescent cell accumulation not only in adipose tissue but also throughout the body (Hohensinner et al., 2018).

However, not all subjects can undergo bariatric surgery or are capable of increased physical activity. Therefore, the pharmacological reduction of senescent cells represents a promising new strategy in the prevention and treatment of obesity and T2DM in more compromised patients.

## 5 Conclusion and future perspectives

The prevalence of obesity and T2DM is steadily increasing worldwide, primarily due to unhealthy lifestyle, an aging population, and a lack of effective measures to reduce risk factors associated with the development and progression of both T2DM and obesity. In addition to its negative impact on the affected individuals, obesity and T2DM lead to a deterioration of population health and impose a rising economic burden on society. The limited efficacy of existing treatments highlights the need to explore new therapeutic options that can address the primary cause of metabolic diseases and prevent their chronic complications. The accumulation of senescent cells in the body plays a key role in the development and progression of obesity, T2DM, and other metabolic complications by contributing to pancreatic β-cell dysfunction, IR, adipose tissue dysfunction, chronic inflammation, and tissue damage due to increased oxidative stress. As cellular senescence is closely linked to the pathogenesis of obesity, T2DM and related complications, targeting senescent cells may be a novel promising strategy of prevention or treatment of these metabolic diseases.

## References

[B1] Abdellatif M. Trummer-Herbst V. Heberle A. M. Humnig A. Pendl T. Durand S. (2022). Fine-tuning cardiac insulin-like growth factor 1 receptor signaling to promote health and longevity. Circulation 145 (25), 1853–1866. 10.1161/CIRCULATIONAHA.122.059863 35616058 PMC9203038

[B2] Aguayo-Mazzucato C. Andle J. Lee T. B. Jr. Midha A. Talemal L. Chipashvili V. (2019). Acceleration of beta cell aging determines diabetes and senolysis improves disease outcomes. Cell Metab. 30 (1), 129–142. 10.1016/j.cmet.2019.05.006 31155496 PMC6610720

[B3] Aguayo-Mazzucato C. van Haaren M. Mruk M. Lee T. B. Jr. Crawford C. Hollister-Lock J. (2017). Beta cell aging markers have heterogeneous distribution and are induced by insulin resistance. Cell Metab. 25 (4), 898–910 e5. 10.1016/j.cmet.2017.03.015 28380379 PMC5471618

[B4] Aguiar-Oliveira M. H. Bartke A. (2019). Growth hormone deficiency: health and longevity. Endocr. Rev. 40 (2), 575–601. 10.1210/er.2018-00216 30576428 PMC6416709

[B5] Alessio N. Acar M. B. Demirsoy I. H. Squillaro T. Siniscalco D. Di Bernardo G. (2020). Obesity is associated with senescence of mesenchymal stromal cells derived from bone marrow, subcutaneous and visceral fat of young mice. Aging (Albany NY) 12 (13), 12609–12621. 10.18632/aging.103606 32634118 PMC7377882

[B6] AlQudah M. Hale T. M. Czubryt M. P. (2020). Targeting the renin-angiotensin-aldosterone system in fibrosis. Matrix Biol. 91-92, 92–108. 10.1016/j.matbio.2020.04.005 32422329 PMC7434656

[B7] Alsalem M. Ellaithy A. Bloukh S. Haddad M. Saleh T. (2024). Targeting therapy-induced senescence as a novel strategy to combat chemotherapy-induced peripheral neuropathy. Support Care Cancer 32 (1), 85. 10.1007/s00520-023-08287-0 38177894

[B8] Althunibat O. Y. Al Hroob A. M. Abukhalil M. H. Germoush M. O. Bin-Jumah M. Mahmoud A. M. (2019). Fisetin ameliorates oxidative stress, inflammation and apoptosis in diabetic cardiomyopathy. Life Sci. 221, 83–92. 10.1016/j.lfs.2019.02.017 30742869

[B9] Armstrong G. T. Kawashima T. Leisenring W. Stratton K. Stovall M. Hudson M. M. (2014). Aging and risk of severe, disabling, life-threatening, and fatal events in the childhood cancer survivor study. J. Clin. Oncol. 32 (12), 1218–1227. 10.1200/JCO.2013.51.1055 24638000 PMC3986385

[B10] Baker D. J. Petersen R. C. (2018). Cellular senescence in brain aging and neurodegenerative diseases: evidence and perspectives. J. Clin. Invest 128 (4), 1208–1216. 10.1172/JCI95145 29457783 PMC5873891

[B11] Barnes P. J. (2017). Senescence in COPD and its comorbidities. Annu. Rev. Physiol. 79, 517–539. 10.1146/annurev-physiol-022516-034314 27959617

[B12] Barzilai N. Crandall J. P. Kritchevsky S. B. Espeland M. A. (2016). Metformin as a tool to target aging. Cell Metab. 23 (6), 1060–1065. 10.1016/j.cmet.2016.05.011 27304507 PMC5943638

[B13] Baur J. A. Pearson K. J. Price N. L. Jamieson H. A. Lerin C. Kalra A. (2006). Resveratrol improves health and survival of mice on a high-calorie diet. Nature 444 (7117), 337–342. 10.1038/nature05354 17086191 PMC4990206

[B14] Besancenot R. Chaligne R. Tonetti C. Pasquier F. Marty C. Lecluse Y. (2010). A senescence-like cell-cycle arrest occurs during megakaryocytic maturation: implications for physiological and pathological megakaryocytic proliferation. PLoS Biol. 8 (9), e1000476. 10.1371/journal.pbio.1000476 20838657 PMC2935456

[B15] Birch J. Gil J. (2020). Senescence and the SASP: many therapeutic avenues. Genes Dev. 34 (23-24), 1565–1576. 10.1101/gad.343129.120 33262144 PMC7706700

[B16] Bodnar A. G. Ouellette M. Frolkis M. Holt S. E. Chiu C. P. Morin G. B. (1998). Extension of life-span by introduction of telomerase into normal human cells. Science 279 (5349), 349–352. 10.1126/science.279.5349.349 9454332

[B17] Bonnet L. Alexandersson I. Baboota R. K. Kroon T. Oscarsson J. Smith U. (2022). Cellular senescence in hepatocytes contributes to metabolic disturbances in NASH. Front. Endocrinol. (Lausanne) 13, 957616. 10.3389/fendo.2022.957616 36072934 PMC9441597

[B18] Bringardner B. D. Baran C. P. Eubank T. D. Marsh C. B. (2008). The role of inflammation in the pathogenesis of idiopathic pulmonary fibrosis. Antioxid. Redox Signal 10 (2), 287–301. 10.1089/ars.2007.1897 17961066 PMC2737712

[B20] Cao Y. Zeng W. Cui Y. Kong X. Wang M. Yu J. (2018). Increased myocardial extracellular volume assessed by cardiovascular magnetic resonance T1 mapping and its determinants in type 2 diabetes mellitus patients with normal myocardial systolic strain. Cardiovasc Diabetol. 17 (1), 7. 10.1186/s12933-017-0651-2 29301529 PMC5755204

[B21] Chen H. Chen H. Liang J. Gu X. Zhou J. Xie C. (2020). TGF-β1/IL-11/MEK/ERK signaling mediates senescence-associated pulmonary fibrosis in a stress-induced premature senescence model of Bmi-1 deficiency. Exp. Mol. Med. 52 (1), 130–151. 10.1038/s12276-019-0371-7 31959867 PMC7000795

[B22] Chen P. Y. Qin L. Li G. Wang Z. Dahlman J. E. Malagon-Lopez J. (2019b). Endothelial TGF-beta signalling drives vascular inflammation and atherosclerosis. Nat. Metab. 1 (9), 912–926. 10.1038/s42255-019-0102-3 31572976 PMC6767930

[B23] Chen Y. Y. Yu X. Y. Chen L. Vaziri N. D. Ma S. C. Zhao Y. Y. (2019a). Redox signaling in aging kidney and opportunity for therapeutic intervention through natural products. Free Radic. Biol. Med. 141, 141–149. 10.1016/j.freeradbiomed.2019.06.012 31199964

[B24] Coelho M. Oliveira T. Fernandes R. (2013). Biochemistry of adipose tissue: an endocrine organ. Arch. Med. Sci. 9 (2), 191–200. 10.5114/aoms.2013.33181 23671428 PMC3648822

[B25] Covarrubias A. J. Kale A. Perrone R. Lopez-Dominguez J. A. Pisco A. O. Kasler H. G. (2020). Senescent cells promote tissue NAD(+) decline during ageing via the activation of CD38(+) macrophages. Nat. Metab. 2 (11), 1265–1283. 10.1038/s42255-020-00305-3 33199924 PMC7908681

[B26] Cree L. M. Patel S. K. Pyle A. Lynn S. Turnbull D. M. Chinnery P. F. (2008). Age-related decline in mitochondrial DNA copy number in isolated human pancreatic islets. Diabetologia 51 (8), 1440–1443. 10.1007/s00125-008-1054-4 18528676

[B27] Cristofalo V. J. Lorenzini A. Allen R. G. Torres C. Tresini M. (2004). Replicative senescence: a critical review. Mech. Ageing Dev. 125 (10-11), 827–848. 10.1016/j.mad.2004.07.010 15541776

[B28] Dai D. F. Chiao Y. A. Marcinek D. J. Szeto H. H. Rabinovitch P. S. (2014). Mitochondrial oxidative stress in aging and healthspan. Longev. Heal. 3, 6. 10.1186/2046-2395-3-6 PMC4013820 24860647

[B29] Demaria M. Ohtani N. Youssef S. A. Rodier F. Toussaint W. Mitchell J. R. (2014). An essential role for senescent cells in optimal wound healing through secretion of PDGF-AA. Dev. Cell 31 (6), 722–733. 10.1016/j.devcel.2014.11.012 25499914 PMC4349629

[B30] de Oliveira Silva T. Lunardon G. Lino C. A. de Almeida Silva A. Zhang S. Irigoyen M. C. C. (2025). Senescent cell depletion alleviates obesity-related metabolic and cardiac disorders. Mol. Metab. 91, 102065. 10.1016/j.molmet.2024.102065 39557194 PMC11636344

[B31] Di Micco R. Krizhanovsky V. Baker D. d'Adda di Fagagna F. (2021). Cellular senescence in ageing: from mechanisms to therapeutic opportunities. Nat. Rev. Mol. Cell Biol. 22 (2), 75–95. 10.1038/s41580-020-00314-w 33328614 PMC8344376

[B32] Dong W. Jia C. Li J. Zhou Y. Luo Y. Liu J. (2022). Fisetin attenuates diabetic nephropathy-induced podocyte injury by inhibiting NLRP3 inflammasome. Front. Pharmacol. 13, 783706. 10.3389/fphar.2022.783706 35126159 PMC8816314

[B33] Du K. Umbaugh D. S. Liuyang W. Jun J. H. Dutta R. K. Oh S. H. (2025). Targeting senescent hepatocytes for treatment of metabolic dysfunction-associated steatotic liver disease and multi-organ dysfunction. Nat. Commun. 16 (1), 3038. 10.1038/s41467-025-57616-w 40155379 PMC11953480

[B34] El Assar M. Alvarez-Bustos A. Sosa P. Angulo J. Rodriguez-Manas L. (2022). Effect of physical activity/exercise on oxidative stress and inflammation in muscle and vascular aging. Int. J. Mol. Sci. 23 (15), 8713. 10.3390/ijms23158713 35955849 PMC9369066

[B35] Feng X. Wang L. Zhou R. Zhou R. Chen L. Peng H. (2023). Senescent immune cells accumulation promotes brown adipose tissue dysfunction during aging. Nat. Commun. 14 (1), 3208. 10.1038/s41467-023-38842-6 37268694 PMC10237528

[B36] Fijany A. Sayadi L. R. Khoshab N. Banyard D. A. Shaterian A. Alexander M. (2019). Mesenchymal stem cell dysfunction in diabetes. Mol. Biol. Rep. 46 (1), 1459–1475. 10.1007/s11033-018-4516-x 30484107

[B37] Foretz M. Guigas B. Bertrand L. Pollak M. Viollet B. (2014). Metformin: from mechanisms of action to therapies. Cell Metab. 20 (6), 953–966. 10.1016/j.cmet.2014.09.018 25456737

[B38] Foretz M. Viollet B. (2015). Therapy: metformin takes a new route to clinical efficacy. Nat. Rev. Endocrinol. 11 (7), 390–392. 10.1038/nrendo.2015.85 26032104 PMC4676264

[B39] Fox C. S. Massaro J. M. Hoffmann U. Pou K. M. Maurovich-Horvat P. Liu C. Y. (2007). Abdominal visceral and subcutaneous adipose tissue compartments: association with metabolic risk factors in the Framingham Heart Study. Circulation 116 (1), 39–48. 10.1161/CIRCULATIONAHA.106.675355 17576866

[B40] Gabbin B. Meraviglia V. Mummery C. L. Rabelink T. J. van Meer B. J. van den Berg C. W. (2022). Toward human models of cardiorenal syndrome *in vitro* . Front. Cardiovasc. Med. 9, 889553. 10.3389/fcvm.2022.889553 35694669 PMC9177996

[B41] Gevaert A. B. Shakeri H. Leloup A. J. Van Hove C. E. De Meyer G. R. Y. Vrints C. J. (2017). Endothelial senescence contributes to heart failure with preserved ejection fraction in an aging mouse model. Circ. Heart Fail 10 (6), e003806. 10.1161/CIRCHEARTFAILURE.116.003806 28611124

[B42] Gomes P. Simao S. Silva E. Pinto V. Amaral J. S. Afonso J. (2009). Aging increases oxidative stress and renal expression of oxidant and antioxidant enzymes that are associated with an increased trend in systolic blood pressure. Oxid. Med. Cell Longev. 2 (3), 138–145. 10.4161/oxim.2.3.8819 20592768 PMC2763239

[B43] Guo W. Pirtskhalava T. Tchkonia T. Xie W. Thomou T. Han J. (2007). Aging results in paradoxical susceptibility of fat cell progenitors to lipotoxicity. Am. J. Physiol. Endocrinol. Metab. 292 (4), E1041–E1051. 10.1152/ajpendo.00557.2006 17148751

[B44] Harrison D. E. Strong R. Sharp Z. D. Nelson J. F. Astle C. M. Flurkey K. (2009). Rapamycin fed late in life extends lifespan in genetically heterogeneous mice. Nature 460 (7253), 392–395. 10.1038/nature08221 19587680 PMC2786175

[B45] Hayflick L. (1965). The limited *in vitro* lifetime of human diploid cell strains. Exp. Cell Res. 37, 614–636. 10.1016/0014-4827(65)90211-9 14315085

[B46] Hemann M. T. Strong M. A. Hao L. Y. Greider C. W. (2001). The shortest telomere, not average telomere length, is critical for cell viability and chromosome stability. Cell 107 (1), 67–77. 10.1016/s0092-8674(01)00504-9 11595186

[B47] Hohensinner P. J. Kaun C. Ebenbauer B. Hackl M. Demyanets S. Richter D. (2018). Reduction of premature aging markers after gastric bypass surgery in morbidly obese patients. Obes. Surg. 28 (9), 2804–2810. 10.1007/s11695-018-3247-3 29693219 PMC6132736

[B48] Hubackova S. Davidova E. Rohlenova K. Stursa J. Werner L. Andera L. (2019). Selective elimination of senescent cells by mitochondrial targeting is regulated by ANT2. Cell Death Differ. 26 (2), 276–290. 10.1038/s41418-018-0118-3 29786070 PMC6329828

[B49] Ibrahim M. M. (2010). Subcutaneous and visceral adipose tissue: structural and functional differences. Obes. Rev. 11 (1), 11–18. 10.1111/j.1467-789X.2009.00623.x 19656312

[B50] Ihm S. H. Moon H. J. Kang J. G. Park C. Y. Oh K. W. Jeong I. K. (2007). Effect of aging on insulin secretory function and expression of beta cell function-related genes of islets. Diabetes Res. Clin. Pract. 77 (Suppl. 1), S150–S154. 10.1016/j.diabres.2007.01.049 17467845

[B51] Joussen A. M. Poulaki V. Le M. L. Koizumi K. Esser C. Janicki H. (2004). A central role for inflammation in the pathogenesis of diabetic retinopathy. FASEB J. 18 (12), 1450–1452. 10.1096/fj.03-1476fje 15231732

[B52] Kahn S. E. Hull R. L. Utzschneider K. M. (2006). Mechanisms linking obesity to insulin resistance and type 2 diabetes. Nature 444 (7121), 840–846. 10.1038/nature05482 17167471

[B53] Kang T. W. Yevsa T. Woller N. Hoenicke L. Wuestefeld T. Dauch D. (2011). Senescence surveillance of pre-malignant hepatocytes limits liver cancer development. Nature 479 (7374), 547–551. 10.1038/nature10599 22080947

[B54] Kim H. W. Shi H. Winkler M. A. Lee R. Weintraub N. L. (2020). Perivascular adipose tissue and vascular perturbation/atherosclerosis. Arterioscler. Thromb. Vasc. Biol. 40 (11), 2569–2576. 10.1161/ATVBAHA.120.312470 32878476 PMC7577939

[B55] Kim M. N. Moon J. H. Cho Y. M. (2021). Sodium-glucose cotransporter-2 inhibition reduces cellular senescence in the diabetic kidney by promoting ketone body-induced NRF2 activation. Diabetes Obes. Metab. 23 (11), 2561–2571. 10.1111/dom.14503 34318973

[B56] Kimura R. Okouchi M. Fujioka H. Ichiyanagi A. Ryuge F. Mizuno T. (2009). Glucagon-like peptide-1 (GLP-1) protects against methylglyoxal-induced PC12 cell apoptosis through the PI3K/Akt/mTOR/GCLc/redox signaling pathway. Neuroscience 162 (4), 1212–1219. 10.1016/j.neuroscience.2009.05.025 19463904

[B57] Kirkland J. L. Dobson D. E. (1997). Preadipocyte function and aging: links between age-related changes in cell dynamics and altered fat tissue function. J. Am. Geriatr. Soc. 45 (8), 959–967. 10.1111/j.1532-5415.1997.tb02967.x 9256849

[B58] Kiss K. Regos E. Rada K. Firneisz G. Baghy K. Kovalszky I. (2020). Chronic hyperglycaemia induced alterations of hepatic stellate cells differ from the effect of TGFB1, and point toward metabolic stress. Pathol. Oncol. Res. 26 (1), 291–299. 10.1007/s12253-018-0458-9 30109568

[B59] Kruglikov I. L. Scherer P. E. (2020). The role of adipocytes and adipocyte-like cells in the severity of COVID-19 infections. Obes. (Silver Spring) 28 (7), 1187–1190. 10.1002/oby.22856 PMC7267593 32339391

[B60] Krupczak-Hollis K. Wang X. Dennewitz M. B. Costa R. H. (2003). Growth hormone stimulates proliferation of old-aged regenerating liver through forkhead box m1b. Hepatology 38 (6), 1552–1562. 10.1016/j.hep.2003.08.052 14647066

[B61] Kuki S. Imanishi T. Kobayashi K. Matsuo Y. Obana M. Akasaka T. (2006). Hyperglycemia accelerated endothelial progenitor cell senescence via the activation of p38 mitogen-activated protein kinase. Circ. J. 70 (8), 1076–1081. 10.1253/circj.70.1076 16864945

[B62] Kulkarni A. S. Brutsaert E. F. Anghel V. Zhang K. Bloomgarden N. Pollak M. (2018). Metformin regulates metabolic and nonmetabolic pathways in skeletal muscle and subcutaneous adipose tissues of older adults. Aging Cell 17 (2), e12723. 10.1111/acel.12723 29383869 PMC5847877

[B63] Kurz D. J. Decary S. Hong Y. Erusalimsky J. D. (2000). Senescence-associated (beta)-galactosidase reflects an increase in lysosomal mass during replicative ageing of human endothelial cells. J. Cell Sci. 113 (Pt 20), 3613–3622. 10.1242/jcs.113.20.3613 11017877

[B64] Le P. Tatar M. Dasarathy S. Alkhouri N. Herman W. H. Taksler G. B. (2025). Estimated burden of metabolic dysfunction-associated steatotic liver disease in US adults, 2020 to 2050. JAMA Netw. Open 8 (1), e2454707. 10.1001/jamanetworkopen.2024.54707 39821400 PMC11742522

[B65] Leasher J. L. Bourne R. R. Flaxman S. R. Jonas J. B. Keeffe J. Naidoo K. (2016). Global estimates on the number of people blind or visually impaired by diabetic retinopathy: a meta-analysis from 1990 to 2010. Diabetes Care 39 (9), 1643–1649. 10.2337/dc15-2171 27555623

[B66] Lefevre C. Chartoire D. Ferraz J. C. Verdier T. Pinteur C. Chanon S. (2021). Obesity activates immunomodulating properties of mesenchymal stem cells in adipose tissue with differences between localizations. FASEB J. 35 (6), e21650. 10.1096/fj.202002046RR 33993539

[B67] Lewis-McDougall F. C. Ruchaya P. J. Domenjo-Vila E. Teoh T. S. Prata L. Cottle B. J. (2019). Aged-senescent cells contribute to impaired heart regeneration. Aging Cell 18 (3), e12931. 10.1111/acel.12931 30854802 PMC6516154

[B68] Liao Y. L. Fang Y. F. Sun J. X. Dou G. R. (2024). Senescent endothelial cells: a potential target for diabetic retinopathy. Angiogenesis 27 (4), 663–679. 10.1007/s10456-024-09943-7 39215875 PMC11564237

[B69] Liu J. Yang J. R. Chen X. M. Cai G. Y. Lin L. R. He Y. N. (2015). Impact of ER stress-regulated ATF4/p16 signaling on the premature senescence of renal tubular epithelial cells in diabetic nephropathy. Am. J. Physiol. Cell Physiol. 308 (8), C621–C630. 10.1152/ajpcell.00096.2014 25567807

[B70] Madonna R. Doria V. Minnucci I. Pucci A. Pierdomenico D. S. De Caterina R. (2020). Empagliflozin reduces the senescence of cardiac stromal cells and improves cardiac function in a murine model of diabetes. J. Cell Mol. Med. 24 (21), 12331–12340. 10.1111/jcmm.15699 32940423 PMC7687009

[B71] Maechler P. Carobbio S. Rubi B. (2006). In beta-cells, mitochondria integrate and generate metabolic signals controlling insulin secretion. Int. J. Biochem. Cell Biol. 38 (5-6), 696–709. 10.1016/j.biocel.2005.12.006 16443386

[B72] Maejima Y. (2019). SGLT2 inhibitors play a salutary role in heart failure via modulation of the mitochondrial function. Front. Cardiovasc Med. 6, 186. 10.3389/fcvm.2019.00186 31970162 PMC6960132

[B73] Martin-Montalvo A. Mercken E. M. Mitchell S. J. Palacios H. H. Mote P. L. Scheibye-Knudsen M. (2013). Metformin improves healthspan and lifespan in mice. Nat. Commun. 4, 2192. 10.1038/ncomms3192 23900241 PMC3736576

[B74] Matloch Z. Cinkajzlova A. Mraz M. Haluzik M. (2018). The role of inflammation in epicardial adipose tissue in heart diseases. Curr. Pharm. Des. 24 (3), 297–309. 10.2174/1381612824666180110102125 29318960

[B75] Mehmel M. Jovanovic N. Spitz U. (2020). Nicotinamide riboside-the current state of research and therapeutic uses. Nutrients 12 (6), 1616. 10.3390/nu12061616 32486488 PMC7352172

[B76] Meng A. Wang Y. Van Zant G. Zhou D. (2003). Ionizing radiation and busulfan induce premature senescence in murine bone marrow hematopoietic cells. Cancer Res. 63 (17), 5414–5419. 14500376

[B77] Miao X. Y. Gu Z. Y. Liu P. Hu Y. Li L. Gong Y. P. (2013). The human glucagon-like peptide-1 analogue liraglutide regulates pancreatic beta-cell proliferation and apoptosis via an AMPK/mTOR/P70S6K signaling pathway. Peptides 39, 71–79. 10.1016/j.peptides.2012.10.006 23116613

[B78] Mills K. F. Yoshida S. Stein L. R. Grozio A. Kubota S. Sasaki Y. (2016). Long-term administration of nicotinamide mononucleotide mitigates age-associated physiological decline in mice. Cell Metab. 24 (6), 795–806. 10.1016/j.cmet.2016.09.013 28068222 PMC5668137

[B79] Mittal N. Siddiqi H. Madamba E. Richards L. Bettencourt R. Ajmera V. (2024). A prospective study on the prevalence of at-risk MASH in patients with type 2 diabetes mellitus in the United States. Aliment. Pharmacol. Ther. 59 (12), 1571–1578. 10.1111/apt.17997 38586922 PMC12425555

[B80] Miyamoto K. Khosrof S. Bursell S. E. Rohan R. Murata T. Clermont A. C. (1999). Prevention of leukostasis and vascular leakage in streptozotocin-induced diabetic retinopathy via intercellular adhesion molecule-1 inhibition. Proc. Natl. Acad. Sci. U. S. A. 96 (19), 10836–10841. 10.1073/pnas.96.19.10836 10485912 PMC17969

[B81] Mosteiro L. Pantoja C. Alcazar N. Marion R. M. Chondronasiou D. Rovira M. (2016). Tissue damage and senescence provide critical signals for cellular reprogramming *in vivo* . Science. 354 (6315), aaf4445. 10.1126/science.aaf4445 27884981

[B82] Munoz-Espin D. Canamero M. Maraver A. Gomez-Lopez G. Contreras J. Murillo-Cuesta S. (2013). Programmed cell senescence during mammalian embryonic development. Cell 155 (5), 1104–1118. 10.1016/j.cell.2013.10.019 24238962

[B83] Nambiar A. Kellogg D. 3rd Justice J. Goros M. Gelfond J. Pascual R. (2023). Senolytics dasatinib and quercetin in idiopathic pulmonary fibrosis: results of a phase I, single-blind, single-center, randomized, placebo-controlled pilot trial on feasibility and tolerability. EBioMedicine 90, 104481. 10.1016/j.ebiom.2023.104481 36857968 PMC10006434

[B84] Nawaz S. S. Siddiqui K. (2022). Plasminogen activator inhibitor-1 mediate downregulation of adiponectin in type 2 diabetes patients with metabolic syndrome. Cytokine x. 4 (1), 100064. 10.1016/j.cytox.2022.100064 35128381 PMC8803603

[B85] Nehme J. Yang D. Altulea A. Varela-Eirin M. Wang L. Hu S. (2023). High dietary protein and fat contents exacerbate hepatic senescence and SASP in mice. FEBS J. 290 (5), 1340–1347. 10.1111/febs.16292 34908245

[B86] Ning Y. C. Cai G. Y. Zhuo L. Gao J. J. Dong D. Cui S. (2013). Short-term calorie restriction protects against renal senescence of aged rats by increasing autophagic activity and reducing oxidative damage. Mech. Ageing Dev. 134 (11-12), 570–579. 10.1016/j.mad.2013.11.006 24291536

[B87] Nordheim E. Geir Jenssen T. (2021). Chronic kidney disease in patients with diabetes mellitus. Endocr. Connect. 10 (5), R151–R159. 10.1530/EC-21-0097 33830068 PMC8111312

[B88] Obesity (2000). Obesity: preventing and managing the global epidemic. Report of a WHO consultation. World Health Organ Tech. Rep. Ser. 894, 1–253. Available online at: https://pubmed.ncbi.nlm.nih.gov/11234459/ 11234459

[B89] Ogrodnik M. Miwa S. Tchkonia T. Tiniakos D. Wilson C. L. Lahat A. (2017). Cellular senescence drives age-dependent hepatic steatosis. Nat. Commun. 8, 15691. 10.1038/ncomms15691 28608850 PMC5474745

[B90] Ogrodnik M. Zhu Y. Langhi L. G. P. Tchkonia T. Kruger P. Fielder E. (2019). Obesity-induced cellular senescence drives anxiety and impairs neurogenesis. Cell Metab. 29 (5), 1061–1077. 10.1016/j.cmet.2018.12.008 30612898 PMC6509403

[B91] Ogurtsova K. da Rocha Fernandes J. D. Huang Y. Linnenkamp U. Guariguata L. Cho N. H. (2017). IDF Diabetes Atlas: global estimates for the prevalence of diabetes for 2015 and 2040. Diabetes Res. Clin. Pract. 128, 40–50. 10.1016/j.diabres.2017.03.024 28437734

[B92] Okabe K. Yaku K. Uchida Y. Fukamizu Y. Sato T. Sakurai T. (2022). Oral administration of nicotinamide mononucleotide is safe and efficiently increases blood nicotinamide adenine dinucleotide levels in healthy subjects. Front. Nutr. 9, 868640. 10.3389/fnut.2022.868640 35479740 PMC9036060

[B93] Ortiz L. A. Lasky J. Hamilton R. F. Jr. Holian A. Hoyle G. W. Banks W. (1998). Expression of TNF and the necessity of TNF receptors in bleomycin-induced lung injury in mice. Exp. Lung Res. 24 (6), 721–743. 10.3109/01902149809099592 9839161

[B94] Ortiz L. A. Lasky J. Lungarella G. Cavarra E. Martorana P. Banks W. A. (1999). Upregulation of the p75 but not the p55 TNF-alpha receptor mRNA after silica and bleomycin exposure and protection from lung injury in double receptor knockout mice. Am. J. Respir. Cell Mol. Biol. 20 (4), 825–833. 10.1165/ajrcmb.20.4.3193 10101016

[B95] Oubaha M. Miloudi K. Dejda A. Guber V. Mawambo G. Germain M. A. (2016). Senescence-associated secretory phenotype contributes to pathological angiogenesis in retinopathy. Sci. Transl. Med. 8 (362), 362ra144. 10.1126/scitranslmed.aaf9440 27797960

[B96] Owen M. R. Doran E. Halestrap A. P. (2000). Evidence that metformin exerts its anti-diabetic effects through inhibition of complex 1 of the mitochondrial respiratory chain. Biochem. J. 348 (Pt 3), 607–614. 10.1042/bj3480607 10839993 PMC1221104

[B97] Palmer A. K. Tchkonia T. LeBrasseur N. K. Chini E. N. Xu M. Kirkland J. L. (2015). Cellular senescence in type 2 diabetes: a therapeutic opportunity. Diabetes 64 (7), 2289–2298. 10.2337/db14-1820 26106186 PMC4477358

[B98] Palmer A. K. Xu M. Zhu Y. Pirtskhalava T. Weivoda M. M. Hachfeld C. M. (2019). Targeting senescent cells alleviates obesity-induced metabolic dysfunction. Aging Cell 18 (3), e12950. 10.1111/acel.12950 30907060 PMC6516193

[B99] Pan X. X. Yao K. L. Yang Y. F. Ge Q. Zhang R. Gao P. J. (2021). Senescent T cell induces Brown adipose tissue “whitening” *via* secreting IFN-γ. Front. Cell Dev. Biol. 9, 637424. 10.3389/fcell.2021.637424 33748126 PMC7969812

[B100] Park S. J. Ahmad F. Philp A. Baar K. Williams T. Luo H. (2012). Resveratrol ameliorates aging-related metabolic phenotypes by inhibiting cAMP phosphodiesterases. Cell 148 (3), 421–433. 10.1016/j.cell.2012.01.017 22304913 PMC3431801

[B101] Patro B. S. Frohlich R. Bohr V. A. Stevnsner T. (2011). WRN helicase regulates the ATR-CHK1-induced S-phase checkpoint pathway in response to topoisomerase-I-DNA covalent complexes. J. Cell Sci. 124 (Pt 23), 3967–3979. 10.1242/jcs.081372 22159421 PMC3244981

[B102] Peng W. Zhou R. Sun Z. F. Long J. W. Gong Y. Q. (2022). Novel insights into the roles and mechanisms of GLP-1 receptor agonists against aging-related diseases. Aging Dis. 13 (2), 468–490. 10.14336/AD.2021.0928 35371594 PMC8947838

[B103] Perry R. J. Samuel V. T. Petersen K. F. Shulman G. I. (2014). The role of hepatic lipids in hepatic insulin resistance and type 2 diabetes. Nature 510 (7503), 84–91. 10.1038/nature13478 24899308 PMC4489847

[B104] Polonis K. Becari C. Chahal C. A. A. Zhang Y. Allen A. M. Kellogg T. A. (2020). Chronic intermittent hypoxia triggers a senescence-like phenotype in human white preadipocytes. Sci. Rep. 10 (1), 6846. 10.1038/s41598-020-63761-7 32321999 PMC7176724

[B105] Pradhan A. D. Manson J. E. Rifai N. Buring J. E. Ridker P. M. (2001). C-reactive protein, interleukin 6, and risk of developing type 2 diabetes mellitus. JAMA 286 (3), 327–334. 10.1001/jama.286.3.327 11466099

[B106] Raicevic B. Jankovic S. (2023). Predictors of gastrointestinal complaints in patients on metformin therapy. Open Med. (Wars). 18 (1), 20230871. 10.1515/med-2023-0871 38045859 PMC10693010

[B107] Reers C. Erbel S. Esposito I. Schmied B. Buchler M. W. Nawroth P. P. (2009). Impaired islet turnover in human donor pancreata with aging. Eur. J. Endocrinol. 160 (2), 185–191. 10.1530/EJE-08-0596 19004984

[B108] Rex N. Melk A. Schmitt R. (2023). Cellular senescence and kidney aging. Clin. Sci. (Lond). 137 (24), 1805–1821. 10.1042/CS20230140 38126209 PMC10739085

[B109] Rohlenova K. Sachaphibulkij K. Stursa J. Bezawork-Geleta A. Blecha J. Endaya B. (2017). Selective disruption of respiratory supercomplexes as a new strategy to suppress her2(high) breast cancer. Antioxid. Redox Signal 26 (2), 84–103. 10.1089/ars.2016.6677 27392540 PMC5206771

[B110] Sabin R. J. Anderson R. M. (2011). Cellular Senescence - its role in cancer and the response to ionizing radiation. Genome Integr. 2 (1), 7. 10.1186/2041-9414-2-7 21834983 PMC3169443

[B111] Salam R. Saliou A. Bielle F. Bertrand M. Antoniewski C. Carpentier C. (2023). Cellular senescence in malignant cells promotes tumor progression in mouse and patient Glioblastoma. Nat. Commun. 14 (1), 441. 10.1038/s41467-023-36124-9 36707509 PMC9883514

[B112] Sasaki N. Itakura Y. Toyoda M. (2020). Rapamycin promotes endothelial–mesenchymal transition during stress-induced premature senescence through the activation of autophagy. Cell Commun. Signal. 18 (1), 43. 10.1186/s12964-020-00533-w 32164764 PMC7069020

[B113] Schafer M. J. White T. A. Iijima K. Haak A. J. Ligresti G. Atkinson E. J. (2017). Cellular senescence mediates fibrotic pulmonary disease. Nat. Commun. 8, 14532. 10.1038/ncomms14532 28230051 PMC5331226

[B114] Serrano M. Lin A. W. McCurrach M. E. Beach D. Lowe S. W. (1997). Oncogenic ras provokes premature cell senescence associated with accumulation of p53 and p16INK4a. Cell 88 (5), 593–602. 10.1016/s0092-8674(00)81902-9 9054499

[B115] Sgalla G. Iovene B. Calvello M. Ori M. Varone F. Richeldi L. (2018). Idiopathic pulmonary fibrosis: pathogenesis and management. Respir. Res. 19 (1), 32. 10.1186/s12931-018-0730-2 29471816 PMC5824456

[B116] Shimokawa I. Higami Y. Tsuchiya T. Otani H. Komatsu T. Chiba T. (2003). Life span extension by reduction of the growth hormone-insulin-like growth factor-1 axis: relation to caloric restriction. FASEB J. 17 (9), 1108–1109. 10.1096/fj.02-0819fje 12692087

[B117] Shioya S. Masuda T. Senoo T. Horimasu Y. Miyamoto S. Nakashima T. (2018). Plasminogen activator inhibitor-1 serves an important role in radiation-induced pulmonary fibrosis. Exp. Ther. Med. 16 (4), 3070–3076. 10.3892/etm.2018.6550 30214528 PMC6125865

[B118] Shivshankar P. Brampton C. Miyasato S. Kasper M. Thannickal V. J. Le Saux C. J. (2012). Caveolin-1 deficiency protects from pulmonary fibrosis by modulating epithelial cell senescence in mice. Am. J. Respir. Cell Mol. Biol. 47 (1), 28–36. 10.1165/rcmb.2011-0349OC 22362388 PMC3402795

[B119] Sis B. Tasanarong A. Khoshjou F. Dadras F. Solez K. Halloran P. F. (2007). Accelerated expression of senescence associated cell cycle inhibitor p16INK4A in kidneys with glomerular disease. Kidney Int. 71 (3), 218–226. 10.1038/sj.ki.5002039 17183247

[B120] Sofue T. Kushida Y. Ozaki T. Moritoki M. Nishijima Y. Ohsaki H. (2018). Tubular cell senescence in the donated kidney predicts allograft function, but not donor remnant kidney function, in living donor kidney transplantation. Am. J. Nephrol. 47 (1), 8–17. 10.1159/000485845 29275400

[B121] Sone H. Kagawa Y. (2005). Pancreatic beta cell senescence contributes to the pathogenesis of type 2 diabetes in high-fat diet-induced diabetic mice. Diabetologia 48 (1), 58–67. 10.1007/s00125-004-1605-2 15624098

[B122] Song K. H. Jeong J. S. Kim M. K. Kwon H. S. Baek K. H. Ko S. H. (2019). Discordance in risk factors for the progression of diabetic retinopathy and diabetic nephropathy in patients with type 2 diabetes mellitus. J. Diabetes Investig. 10 (3), 745–752. 10.1111/jdi.12953 PMC6497586 30300472

[B123] Sookoian S. Pirola C. J. (2011). Metabolic syndrome: from the genetics to the pathophysiology. Curr. Hypertens. Rep. 13 (2), 149–157. 10.1007/s11906-010-0164-9 20957457

[B124] Soriani A. Zingoni A. Cerboni C. Iannitto M. L. Ricciardi M. R. Di Gialleonardo V. (2009). ATM-ATR-dependent up-regulation of DNAM-1 and NKG2D ligands on multiple myeloma cells by therapeutic agents results in enhanced NK-cell susceptibility and is associated with a senescent phenotype. Blood 113 (15), 3503–3511. 10.1182/blood-2008-08-173914 19098271

[B125] Supale S. Thorel F. Merkwirth C. Gjinovci A. Herrera P. L. Scorrano L. (2013). Loss of prohibitin induces mitochondrial damages altering beta-cell function and survival and is responsible for gradual diabetes development. Diabetes 62 (10), 3488–3499. 10.2337/db13-0152 23863811 PMC3781460

[B126] Tchkonia T. Morbeck D. E. Von Zglinicki T. Van Deursen J. Lustgarten J. Scrable H. (2010). Fat tissue, aging, and cellular senescence. Aging Cell 9 (5), 667–684. 10.1111/j.1474-9726.2010.00608.x 20701600 PMC2941545

[B127] Tencerova M. Frost M. Figeac F. Nielsen T. K. Ali D. Lauterlein J. L. (2019). Obesity-associated hypermetabolism and accelerated senescence of bone marrow stromal stem cells suggest a potential mechanism for bone fragility. Cell Rep. 27 (7), 2050–2062. 10.1016/j.celrep.2019.04.066 31091445

[B128] Trnovska J. Svoboda P. Pelantova H. Kuzma M. Kratochvilova H. Kasperova B. J. (2021). Complex positive effects of SGLT-2 inhibitor empagliflozin in the liver, kidney and adipose tissue of hereditary hypertriglyceridemic rats: possible contribution of attenuation of cell senescence and oxidative stress. Int. J. Mol. Sci. 22 (19), 10606. 10.3390/ijms221910606 34638943 PMC8508693

[B129] Tsuji S. Minami S. Hashimoto R. Konishi Y. Suzuki T. Kondo T. (2022). SARS-CoV-2 infection triggers paracrine senescence and leads to a sustained senescence-associated inflammatory response. Nat. Aging 2 (2), 115–124. 10.1038/s43587-022-00170-7 37117754 PMC10154207

[B130] Vacurova E. Trnovska J. Svoboda P. Skop V. Novosadova V. Reguera D. P. (2022). Mitochondrially targeted tamoxifen alleviates markers of obesity and type 2 diabetes mellitus in mice. Nat. Commun. 13 (1), 1866. 10.1038/s41467-022-29486-z 35387987 PMC8987092

[B131] Vacurova E. Vlachova E. Stursa J. Bohacova K. Havrlantova T. Skop V. (2025). Targeting mitochondrial integrity as a new senolytic strategy. Aging Dis. 10.14336/AD.2024.1100 PMC12539531 39965253

[B132] van Deursen J. M. (2014). The role of senescent cells in ageing. Nature 509 (7501), 439–446. 10.1038/nature13193 24848057 PMC4214092

[B133] Verzola D. Gandolfo M. T. Gaetani G. Ferraris A. Mangerini R. Ferrario F. (2008). Accelerated senescence in the kidneys of patients with type 2 diabetic nephropathy. Am. J. Physiol. Ren. Physiol. 295 (5), F1563–F1573. 10.1152/ajprenal.90302.2008 18768588

[B134] Villaret A. Galitzky J. Decaunes P. Esteve D. Marques M. A. Sengenes C. (2010). Adipose tissue endothelial cells from obese human subjects: differences among depots in angiogenic, metabolic, and inflammatory gene expression and cellular senescence. Diabetes 59 (11), 2755–2763. 10.2337/db10-0398 20713685 PMC2963533

[B135] Wadden T. A. Chao A. M. Machineni S. Kushner R. Ard J. Srivastava G. (2023). Tirzepatide after intensive lifestyle intervention in adults with overweight or obesity: the SURMOUNT-3 phase 3 trial. Nat. Med. 29 (11), 2909–2918. 10.1038/s41591-023-02597-w 37840095 PMC10667099

[B136] Wang C. Maddick M. Miwa S. Jurk D. Czapiewski R. Saretzki G. (2010). Adult-onset, short-term dietary restriction reduces cell senescence in mice. Aging (Albany NY) 2 (9), 555–566. 10.18632/aging.100196 20844316 PMC2984605

[B137] Wang Q. Wang J. Wang P. Wang L. Jia L. Ling X. (2019). Glycemic control is associated with atrial structural remodeling in patients with type 2 diabetes. BMC Cardiovasc Disord. 19 (1), 278. 10.1186/s12872-019-1249-2 31791258 PMC6889664

[B138] Wang R. Yu Z. Sunchu B. Shoaf J. Dang I. Zhao S. (2017). Rapamycin inhibits the secretory phenotype of senescent cells by a Nrf2-independent mechanism. Aging Cell 16 (3), 564–574. 10.1111/acel.12587 28371119 PMC5418203

[B139] Wiley C. D. Campisi J. (2021). The metabolic roots of senescence: mechanisms and opportunities for intervention. Nat. Metab. 3 (10), 1290–1301. 10.1038/s42255-021-00483-8 34663974 PMC8889622

[B140] Wilkinson E. Waqar M. Sinclair A. Randhawa G. (2016). Meeting the challenge of diabetes in ageing and diverse populations: a review of the literature from the UK. J. Diabetes Res. 2016, 8030627. 10.1155/2016/8030627 27830158 PMC5086503

[B141] Wissler G. E. O. Misra A. Netto J. M. E. Tchkonia T. Kirkland J. L. (2021). Strategies for late phase preclinical and early clinical trials of senolytics. Mech. Ageing Dev. 200, 111591. 10.1016/j.mad.2021.111591 34699859 PMC8627448

[B142] Xia Y. Jin J. Sun Y. Kong X. Shen Z. Yan R. (2024). Tirzepatide's role in targeting adipose tissue macrophages to reduce obesity-related inflammation and improve insulin resistance. Int. Immunopharmacol. 143 (Pt 2), 113499. 10.1016/j.intimp.2024.113499 39471690

[B143] Xu M. Palmer A. K. Ding H. Weivoda M. M. Pirtskhalava T. White T. A. (2015). Targeting senescent cells enhances adipogenesis and metabolic function in old age. Elife 4, e12997. 10.7554/eLife.12997 26687007 PMC4758946

[B144] Xu M. Pirtskhalava T. Farr J. N. Weigand B. M. Palmer A. K. Weivoda M. M. (2018). Senolytics improve physical function and increase lifespan in old age. Nat. Med. 24 (8), 1246–1256. 10.1038/s41591-018-0092-9 29988130 PMC6082705

[B145] Yoshino J. Mills K. F. Yoon M. J. Imai S. (2011). Nicotinamide mononucleotide, a key NAD(+) intermediate, treats the pathophysiology of diet- and age-induced diabetes in mice. Cell Metab. 14 (4), 528–536. 10.1016/j.cmet.2011.08.014 21982712 PMC3204926

[B146] Younossi Z. M. Golabi P. Price J. K. Owrangi S. Gundu-Rao N. Satchi R. (2024). The global epidemiology of nonalcoholic fatty liver disease and nonalcoholic steatohepatitis among patients with type 2 diabetes. Clin. Gastroenterol. Hepatol. 22 (10), 1999–2010 e8. 10.1016/j.cgh.2024.03.006 38521116

[B147] Yousefzadeh M. J. Zhu Y. McGowan S. J. Angelini L. Fuhrmann-Stroissnigg H. Xu M. (2018). Fisetin is a senotherapeutic that extends health and lifespan. eBioMedicine 36, 18–28. 10.1016/j.ebiom.2018.09.015 30279143 PMC6197652

[B19] Zhang D. Lu H. Chen Z. Wang Y. Lin J. Xu S. (2017). High glucose induces the aging of mesenchymal stem cells via Akt/mTOR signaling. Mol. Med. Rep. 16 (2), 1685–1690. 10.3892/mmr.2017.6832 28656269 PMC5562095

[B148] Zheng Z. Bian Y. Zhang Y. Ren G. Li G. (2020). Metformin activates AMPK/SIRT1/NF-κB pathway and induces mitochondrial dysfunction to drive caspase3/GSDME-mediated cancer cell pyroptosis. Cell Cycle 19 (10), 1089–1104. 10.1080/15384101.2020.1743911 32286137 PMC7217368

[B149] Zhong W. L. Zou G. L. Gu J. Q. Zhang J. (2010). L-arginine attenuates high glucose-accelerated senescence in human umbilical vein endothelial cells. Diabetes Res. Clin. P. R. 89 (1), 38–45. 10.1016/j.diabres.2010.03.013 20398956

[B150] Zhou L. Chen X. Lu M. Wu Q. Yuan Q. Hu C. (2019). Wnt/β-catenin links oxidative stress to podocyte injury and proteinuria. Kidney Int. 95 (4), 830–845. 10.1016/j.kint.2018.10.032 30770219 PMC6431566

[B151] Zhu F. Li Y. Zhang J. Piao C. Liu T. Li H. H. (2013). Senescent cardiac fibroblast is critical for cardiac fibrosis after myocardial infarction. PLoS One 8 (9), e74535. 10.1371/journal.pone.0074535 24040275 PMC3770549

[B152] Zhu X. Zhang C. Liu L. Xu L. Yao L. (2024). Senolytic combination of dasatinib and quercetin protects against diabetic kidney disease by activating autophagy to alleviate podocyte dedifferentiation via the Notch pathway. Int. J. Mol. Med. 53 (3), 26. 10.3892/ijmm.2024.5350 38240118 PMC10852012

[B153] Zhu Y. Tchkonia T. Pirtskhalava T. Gower A. C. Ding H. Giorgadze N. (2015). The Achilles' heel of senescent cells: from transcriptome to senolytic drugs. Aging Cell 14 (4), 644–658. 10.1111/acel.12344 25754370 PMC4531078

[B154] Zizka O. Haluzik M. Jude E. B. (2024). Pharmacological treatment of obesity in older adults. Drugs Aging 41 (11), 881–896. 10.1007/s40266-024-01150-9 39514148 PMC11554829

